# Spasm of the Eye Muscles

**Published:** 1881-02

**Authors:** Julian J. Chisolm

**Affiliations:** Professor of Eye and Ear Diseases in the University of Maryland, Surgeon in charge of the Presbyterian Eye and Ear Charity Hospital of Baltimore, &c.


THE
### Independent Practitioner.

Vol. II.——*February, 1881.*——No. 2.

ARTICLE I.

### SPASM OF THE INTRA-OCULAR EYE MUSCLES; A FREQUENT CAUSE OF ANNOYING AND SERIOUS EYE DEFECTS.

BY JULIAN J. CHISOLM, M. D.,

Professor of Eye and Ear Diseases in the University of Maryland, Surgeon in charge of the Presbyterian Eye and Ear Charity Hospital of Baltimore, &c.

Many eye troubles are explained on the basis of nerve straining, if there be no visible evidences of inflammation present. At least, one would suppose this theory to be of wide acceptation, from the number of patients who come to specialists with such a statement from their family physician. Such cases are usually those of children, driven by ambition, or otherwise, to too much study, who complain of painful eyes, when to all ordinary examination they exhibit no redness nor visible weakness of any kind. As the eye discomfort only comes on during the school session, and no complaints are made during holidays, want of application is the plausible explanation of parents; the teacher calls the complaint by the stronger term of laziness, and where, from constant ailing, the family doctor is called in, he can see no surface evidence of eye disease and hence calls it straining of the optic nerve.

We are all too much led away with the belief that eye work is exclusively a nerve work, and that study is an eye nerve tax; forgetting that as far as the retina and optic nerve are concerned, the eye is in full use during our waking hours, whether impressed by pictures at near or far points. The image made on the retina by a landscape, or the retinal picture of a printed word, is an irritation of a peculiar nature, transmitted to the brain through the optic nerve fibres for interpretation, and hence very properly comes under the general classification of nerve work. To the eye itself the optic nerve work is the same in viewing either near or distant pictures; but when eye activity is considered, these two acts of near and far seeing differ widely. In enjoying the sight of distant objects, the eye is in a passive state as regards certain of its important parts. When viewing near things, whether large or small, these very parts, formerly at rest, are brought into great activity, hence reading is a greater effort with the eye than is sight seeing. The special function involved in seeing near things is called the accommodative act, or the preparation of the crystalline lens to focus light coming from objects placed near to the person. This is a muscular act with which the retina and optic nerve have nothing immediately to do, and therefore in reading or writing they are not called upon to be strained.

To get a wholesome impression of the structure and functions of this very important visual apparatus. we must commence by saying that the eye-ball is a hollow body, especially prepared to contain, protect and nourish a peculiar organ which is placed within it, called the retina. The retina is as much an organ of the living body as is the liver, heart or kidney. Each of these large organs have their coverings, blood-vessels and nerves, and so has the retina. No one would call the liver the terminal expansion of the pneumogastric nerve, nor the heart the terminal development of the cardiac nerves, and therefore, the retina cannot be called the terminal expansion of the optic nerve. The retina, as a most intricate and very important organ, has an entire nerve to itself. The optic nerve, one of the largest of the cranial nerves, with its myriad of nerve fibres, covers the whole inner face of the visual organ, and forms the bond of intimate union between each of the thousands of retinal elements and the central controlling organ, the brain. In the living, animal this organ with its intimate and intricate nerve connections, is ever at work when the pupil is exposed to light. Light is its normal stimulus, and under its influence the retinal elements are undergoing constant changes, whether the light be transmitted from distant or near objects.

In reading, sewing or writing, the adaptation of the lens for concentrating light upon the retina is effected by means of the ciliary muscle. When this intra-ocular muscle contracts, it increases the curvature of the crystalline lens and makes it a more powerful condensing medium. It needs a more powerful lens to focus the divergent rays of light from a near object than the parallel rays from a distant reflecting surface. When one is using the eye for seeing near things, with the lens made stronger by increasing its convexity, this change of shape in the lens requires the continued action of the ciliary muscle, a circular muscle located near the lens and within the eye. Reading is, therefore, a muscular work as well as a nerve work. The ciliary muscle although a very small one, possesses all the attributes of the other muscles of the body. It can contract and relax, it can work long and become tired, and it can express fatigue by soreness or pain. It can also become irritable when over-worked, and show a disposition to keep up an abnormal contraction which in all muscles is called spasm. This irritable contraction of the muscle, which may be pushed to a condition rendering voluntary relaxation impossible, seems to be brought about rather by excessive action than by long continued use.

There are conditions of the eye ball which seem to induce this muscular irregularity. Eye-balls, although made after a general plan, and even in accordance with a well conceived model, vary in size and also in shape. Some eyes, although of perfect shape, are larger than others. In the varied sizes of the eye-ball we find no element of weakness; as an eye must work perfectly whether large or small, provided all of its constituent parts are nicely adjusted and bear a fixed relation to each other. This is no longer the case when the eye-ball is changed in shape and deviates from the spherical form, especially if the eye-ball should be congenitally flattened in its antero-posterior diameter. This flattening causes the retina to be brought nearer the lens, which must then shorten its focus to make perfect pictures on this sensitive surface. Without a sharp focusing no well defined picture of the object looked at is shown upon the retina, and vision would be as if through a fog, clouding more or less the object brought before our eyes.

As far as any muscular activity of the normal eye is concerned, it should be in a perfectly passive state when viewing distant objects:

for in a perfectly formed eye the lens, unaided by muscular contraction, focuses distant light accurately upon the retina, which is at all times prepared to receive images. Should the eye be not properly shaped (or as it is termed, emmetropic, capable of measuring objects clearly at the most varied distances,) but be flattened in the direction of its antero posterior diameter, (a condition known as hypermetropia, an eye that measures more easily distant than near things) then the ever ready retina is approached so near to the lens by this flattened form of the eye-ball that it cannot receive a clear impression of any object unless the lens thickens and increases its magnifying power so as to shorten its focus. This adjustment of the lens to a shorter focus, so that the distant object can be sharply seen, is a muscular act, and indicates a degree of intra ocular muscular work, which is always in inverse proportion to the extent of the eye shortening. Such a short eye is ever active when open, and is only at rest when the individual sleeps.

In a well shaped eye, all approaching objects, to be clearly seen, call for a progressive muscular activity. Instinctively the retina seems to abhor a blurred image, and hence keeps the ciliary muscle ever at work, so that the lens shall always be adjusted to focus sharply the light coming from any object in the direction of which our eyes are turned. It is only the sharp image that the retina accepts. The pupilary opening cannot keep out light coming from every conceivable distance and from every reflecting surface, all of which passes through the lens and must make imperfect shadows on the receiving sensitive surface. It is only the sharply focused image that the retina recognizes. If two objects are held up in line, one foot apart, and the eye directed at first to one and then to the other, it will be found that both cannot be clearly seen simultaneously. If a lady should try to read, with a veil over the face, when she sees the print the intervening veil fades away: and should she force the eyes to see the meshes of the veil the characters in the book are at once lost to sight.

If the perfectly formed eye be passive, for distant vision, it requires full action of the ciliary muscle in such a good eye to see well at a short distance; and as long as such an eye is used for reading, sewing or writing the ciliary muscle is constantly at work. This function of accommodation, in a well formed eye and in a healthy person, can be kept up many hours without sensation of fatigue. Should the muscular system be enfeebled by acute disease, then the eyes soon tire in reading, as under similar conditions the muscles of the limbs would give out in walking. Both are muscular efforts and will cause pain if forced, when the body has been enfeebled. This condition explains the reason why children resuming school after an attack of measles, scarlet fever or diphtheria, find such difficulty in carrying on the studies that gave no trouble before the febrile attack. Where health and strength is required for bodily work the eyes prove themselves fully equal to their share of the labor.

In eyes that are flat, as in the hypermetropic eye, the passive lens focuses behind the retina. Good sight, ever sought by the eye, requires light to be focused upon the retina; hence in flat eyes the ciliary muscle is called upon at all times to do some of its accommodating work. When near sight is required and the usual accommodation of perfect eyes is demanded by the flat one, some of this muscular accommodation is found already used up in distant seeing and the remainder must be overtaxed to ensure a satisfactory focusing. This over exertion, if long continued, naturally fatigues, causing pain over the brow and in the eye. This pain or sense of weariness is a measure of muscular fatigue, and is known by the name of asthenopia. It is not an evidence of nerve straining, and in fact has nothing to do with the optic nerve or retina. When such a muscular effort is only needed for a short time, with a good interval of rest, the feeling of painful weariness is not induced, and any latent fatigue passes off before the eye is called into renewed activity. Should the eye work be increased and the intervals of rest be much shortened, pain is occasioned. This irritation induces the eye to water, it becomes sensitive to strong light and looks injected. Often the muscle, when weary, gives out, and by its relaxation ceases to focus. This condition of the lens from relaxation of the ciliary muscles, accompanied as it is by a relaxation of the internal rectus or converging muscle of the eye, ensures a blurring of the near work. If it be reading, the letters run together and become illegible. Closing the eyes for a few minutes will enable them to see clearly again, but only to be followed by a renewal of the muscular relaxation and a second blurring of the page. A few repetitions of this and the near work has to be abandoned for the time, that the eye muscular rest, so needful may be taken.

There are other cases, however, in which the irritated muscles instead of relaxing, become more irritable and assume nearly a permanent condition of contraction; a regular muscular spasm. This allows the lens to be kept permanently in so highly convexed a state as to fit it only for near work. With such an eye, distant objects become blurred and the individual assumes all of the conditions of a near sighted person. He can no longer recognize friends across the street, and when reading holds the book very near to his face. A near-sighted glass improves the distant vision of such a person, while its use enhances much the danger of eye disease. This condition is very properly called *ciliary spasm* and indicates a very unwholesome state of muscular activity. This irregular contraction is not often found in the eyes of adults. It belongs more especially to the eyes of school children, and is now recognized as the way station to that serious and permanent organic disease of the eyes, called myopia, or near sightedness.

Hypermetropia, or flat eyes in varied degrees, are so often found in children that it may nearly be called the normal condition of the young growing race. It is only when the higher grades of eye flattening is met with, that the over exertion of the eyes is severely felt, and this spasm of the accommodative muscle is met with. Yet every ambitious child at school is liable to have it, and therefore every appearance of acquiring near sightedness in a child should be carefully examined into by some one familiar with eye diseases. If a strong solution of atropia be inserted into such a suspected eye, and under its influence the largely dilated pupil enables the child to see better distant objects and especially when distant vision is made perfect by use of magnifying glasses, the ciliary spasm will be clearly proved. The convex lens with which the patient sees best distant objects, when the eye is atropanized, measures the degree of flattening of the eye ball. The spectacles, if worn for all near work, will take away all effort and prevent any return of the ciliary spasm. A few cases appended to this article in illustration, will explain the condition of ciliary spasm, to which I desire to draw attention.

1ST. CASE, H. F., aged 14, was operated upon by me, 3 years since, for convergent squint. The internal rectus muscle of each eye was divided, and the eye made perfectly straight. No one now looking at the boy would suppose that the eyes had ever been any other than perfect, so completely has the deformity been corrected. At the time of former treatment he was ordered convex glasses, with which he could then read very well. He is now advanced in school, and for the past three months has been working hard at his books. During this period he has acquired the habit of reading with the book held 4 inches from his face, using, at the same time the convex glass. From the proximity of the book, it looks as if his eye already foçused too much, and that the magnifying glasses were not needed ; and yet he cannot see without them. Without the glasses he reads No. 2 of Jæger's Test Types with the left eye, and No. 16 only with the right eye. This shows that his work is done by one eye alone, the right being visibly open, but not aiding him in his studies. Remembering that a former examination proved him to have a flat eye which had formerly caused him to squint, I felt that the apparent near-sightedness might be wholly fictitious, and that it may depend upon a spasm of the ciliary muscle,with a lens held thereby in an excessively convex shape. The need of the magnifying spectacles to enlarge the image of letters, also strengthened the belief that there had been no elongation of the eye ball, to explain his present very near vision in reading. His distant vision was 15-70 of what is normal, needing 1-15 concave glasses for its correction. With a ten inch optometer, his range of accomodation was from ½ the nearest point, to ⅛ the most distant, also verifying the apparent necessity, for concave glasses. Knowing from experience in many similar cases, that the action of a strong mydriatic would throw much light upon the diagnosis, I put into the eyes, several drops of a solution of Duboisin. I preferred this preparation because its action would be prompt, and not so long kept up as would be the instillation of the usual Sulphate of Atropia drop of 4 grs. ℥ 1 of water. An examination of the eye one hour after, the instillation showed a widely dilated pupil. The printed page was now a perfect blank to him. In viewing the test types placed at 15 ft. he could sèe none of them ; the near-sighted glass which had sharpened distant vision, being now no help whatever. With the trial lenses the strength of the magnifying glass was increased from 1-24+ the spectacle with which he had been studying, to 1-5+which gave him the sharpest vision. This is as strong a glass as an old man of 80 requires. The relaxation induced by the solution of Duboisin showed the case to be one of excessive spasm of the ciliary muscle, in an eye that was very flat and which needed very strong convex spectacles for its correction.

2ND CASE, Miss S. aged 12, has complained of painful near-sight for some weeks. She had measles one year since : prior to that attack she was a strong healthy girl, never complaining of her eyes. Now she has constantly headaches, with nausea, and by her frequent moping and listlessness has made her parents very uneasy. They have noticed that of late she has become so very near-sighted that she actually buries her face in the book when reading; and they have imagined that the condition of her eyes has something to do with the constant headaches complained of, because reading for a very short time will often put the child in bed. When examined by me I found the eyes very sensitive to light, slightly injected, and watery. In testing them for distance by the test Types, the letters that she ought to read at one hundred feet could be seen only at 12 feet, giving her 12-100 of perfect distant vision. Near-sighted glasses were a decided improvement to her. The very fine print she could not make out; No. 6 was the best she could do. With the 10 in. optometer, her most distant point for the left eye was 3 inches, and for the right 2 inches. This was an anomalous condition which pointed at once to ciliary spasm as the cause of her near sightedness. A solution of the sulphate of atropia, grs. 4 to ℥ 1 of water, was prescribed, to be used freely in the eyes twice a day, with instructions to return the child for examination in three days. When next seen all headache had vanished, and distant sight was tolerably good. She could now make out number 20 when standing 15 feet from the Test Types, instead of 100 as formerly; and by means of a 36 inch convex lens could read No. 12 at 12 inches, which is equivalent to perfect distant vision. As she complained of being easily fatigued, tonics were prescribed, and the drops of Atropia in the eyes were continued once a day. When two weeks had expired I thought that the Atropia solution could be discontinued, as the ciliary spasm seemed to have passed off, and the headaches and nausea had disappeared with them. The old eye trouble soon returned however, and the drops had to be resumed. In addition, the magnifying glasses were prescribed for constant use, so as to do away with the necessity of using the ciliary muscles, and allow them perfect rest.

These cases are quite typical of the trouble to which I desire to call attention. It is one which induces foggy distant sight, accompanied by painful eyes, often with headaches and weariness of the brow. It is often met with among school children, either from over study or after attacks of any of the acute exanthemata, as Measles or Scarlet-fever. If not recognized in these children, this ciliary spasm will not only establish the fictitious near-sightedness from excessive convexity of the crystalline lens, but will eventually bring about a distention of the eye ball, with yielding of the posterior walls, in the vicinity of the optic nerve entrance and yellow spot of Sœmmering. This yielding of the eye walls establishes a permanent organic myopia, always a serious condition.

It is not exclusively in children, however, that this ciliary spasm is met with. At times we are called upon to correct the irritation in adults, as the following case will show.

Mrs. R., aged 35, married, and with a large family, has been troubled with her eyes for one year. Living a luxurious life, they have not been overtaxed, although she is always engaged in reading, fancy work, &c. Her frequent complaint is headache, which she refers to as a family failing: any use of her eyes at times will much aggravate this trouble. She reads fine print with some difficulty and has only $\frac{3}{4}$ of good distant vision. A 1-30 inch near-sighted glass clears up the distant object and gives her sharp and perfect sight. With the 10 inch optometer she reads at 5 inches, her nearest point and 8 inches the farthest, which, when her age of 35 is taken into consideration, shows the anomaly of ciliary spasm. The 5 inch point for near reading with the optometer, at her age, should allow her to read at ten inches at least, for distance. Under the experimental drop of a strong atropia solution her distant vision remained as before. It was no longer cleared up, however, by the near-sighted glass, which, on the contrary, blurred it the more. With a 1-42+glass distant vision became distinct, and perfect vision was restored. The constant use of the convex glass for all eye work will prevent the spasmodic trouble from again appearing.

Such cases of ciliary spasm are frequently mistaken for what they seem to be, but are not, cases of myopia. If the Test Types and trial glasses be alone used by physicians for the correction of errors of refraction, especially in school children, one will be often led into error in the application of concave glasses, when convex glasses are actually needed. The ophthalmologist can detect these errors of refraction by the ophthalmoscopic examination, but to be an expert in the use of this instrument necessitates every day practice. Those who are familiar with the working of the optometer will find it a useful aid in strengthening suspicions of eye defects, when used in connection with Test Types and Trial Glasses. Once suspicions are aroused, by the rapidly growing near-sightedness, especially in children, a strong mydriatic will alone solve the doubt, and can safely be used. This eye drop, which completely relaxes the ciliary muscle, will show conclusively to what order of functional or organic troubles the defect in seeing belongs. It may be necessary to use frequently the strong solution of Atropia or Duboisin before the ciliary spasm altogether relaxes. One must not be satisfied, therefore, with a single application only. If no muscular spasm exist no harm is done by the application; and should there be irritability and irregular excessive contraction of the eye muscles, complete relaxation will be of great comfort in removing headache and sensations of eye fatigue. It is also the best preparation for the wearing of magnifying glasses, which are to prevent in future the over use of the intra-ocular muscles.

